# Human-machine interfaces based on EMG and EEG applied to robotic systems

**DOI:** 10.1186/1743-0003-5-10

**Published:** 2008-03-26

**Authors:** Andre Ferreira, Wanderley C Celeste, Fernando A Cheein, Teodiano F Bastos-Filho, Mario Sarcinelli-Filho, Ricardo Carelli

**Affiliations:** 1 Department of Electrical Engineering, Federal University of Espirito Santo, Av. Fernando Ferrari, 514, 29075-910, Vitoria-ES, Brazil; 2 Institute of Automatics, National University of San Juan, Av. San Martin, 1109-Oeste, 5400, San Juan, Argentina

## Abstract

**Background:**

Two different Human-Machine Interfaces (HMIs) were developed, both based on electro-biological signals. One is based on the EMG signal and the other is based on the EEG signal. Two major features of such interfaces are their relatively simple data acquisition and processing systems, which need just a few hardware and software resources, so that they are, computationally and financially speaking, low cost solutions. Both interfaces were applied to robotic systems, and their performances are analyzed here. The EMG-based HMI was tested in a mobile robot, while the EEG-based HMI was tested in a mobile robot and a robotic manipulator as well.

**Results:**

Experiments using the EMG-based HMI were carried out by eight individuals, who were asked to accomplish ten eye blinks with each eye, in order to test the eye blink detection algorithm. An average rightness rate of about 95% reached by individuals with the ability to blink both eyes allowed to conclude that the system could be used to command devices. Experiments with EEG consisted of inviting 25 people (some of them had suffered cases of meningitis and epilepsy) to test the system. All of them managed to deal with the HMI in only one training session. Most of them learnt how to use such HMI in less than 15 minutes. The minimum and maximum training times observed were 3 and 50 minutes, respectively.

**Conclusion:**

Such works are the initial parts of a system to help people with neuromotor diseases, including those with severe dysfunctions. The next steps are to convert a commercial wheelchair in an autonomous mobile vehicle; to implement the HMI onboard the autonomous wheelchair thus obtained to assist people with motor diseases, and to explore the potentiality of EEG signals, making the EEG-based HMI more robust and faster, aiming at using it to help individuals with severe motor dysfunctions.

## Background

Electro-biological signals have become the focus of several research institutes, probably stimulated by the recent findings in the areas of cardiology, muscle physiology and neuroscience, by the availability of more efficient and cheaper computational resources, and by the increasing knowledge and comprehension about motor dysfunctions [1,2].

Electrical signals coming from different parts of the human body can be used as command signals for controlling mechanical systems. However, it is necessary that the individual in charge of controlling such devices be able to intentionally generate such signals. It is also necessary that the interface adopted (the Human-Machine Interface – HMI) can "understand" and process such signals, setting the command that better fits the wish of the individual. Then, an HMI can be used to improve the capacity of movement of individuals with motor dysfunctions, using, for example, a robotic wheelchair to carry them.

Many electro-biological signals can be used in connection with HMIs. Some of the more commonly adopted signals are the Electro-Myographic (EMG) signal, the Electro-Oculographic (EOG) signal and the Electro-Encephalographic (EEG) signal. This work presents results related to the use of EMG and EEG signals. The use of EOG signal is still incipient in the studies we have developed so far.

**Figure 1:**
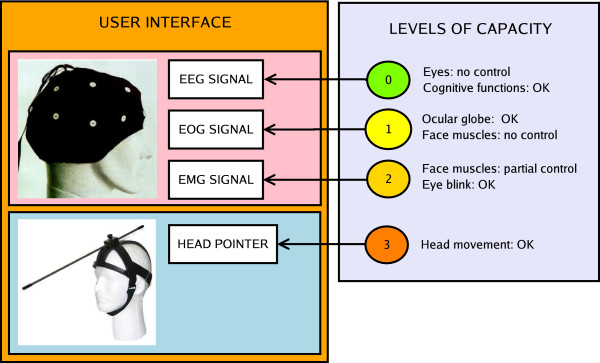
Signals adopted in different Human-Machine Interfaces, and the corresponding levels of capacity.

EMG signals are generated by neuromuscular activity, with signal levels varying from 100 *μV *to 90 *mV *with frequency ranging from DC to 10 *kHz*. Such signals have a standard behavior, which is an important feature to take into account when designing an HMI interface to link an individual with motor dysfunction and a mechanical device. Furthermore, the signal level corresponding to EMG signals is higher when compared to the level corresponding to EEG signals, thus being easier to discriminate its level. In other words, if the individual using the HMI generates normal EMG signals, this kind of signal should be adopted. However, there are some problems inherent to the use of EMG signals. Considering that the assisting technology we deal with in this work is also directed to people with neuromotor disabilities, some muscle spasms, for example, can take place, which represent a serious problem (unless the HMI is robust enough to reject such disturbances) when using EMG signals to control mechanical devices. Severe neuromotor injuries can also cause loss of muscle mobility, which makes impossible to use any kind of EMG-based control to assist individuals with such diseases. Thus, other communication channels (in this scenario other electro-biological signals) should be explored to avoid this kind of problem. As presented in Figure 1, brain signals can be a good solution when EMG and EOG signals are not available, as when assisting individuals with muscle spasms or locked in syndrome [3].

**Figure 2:**
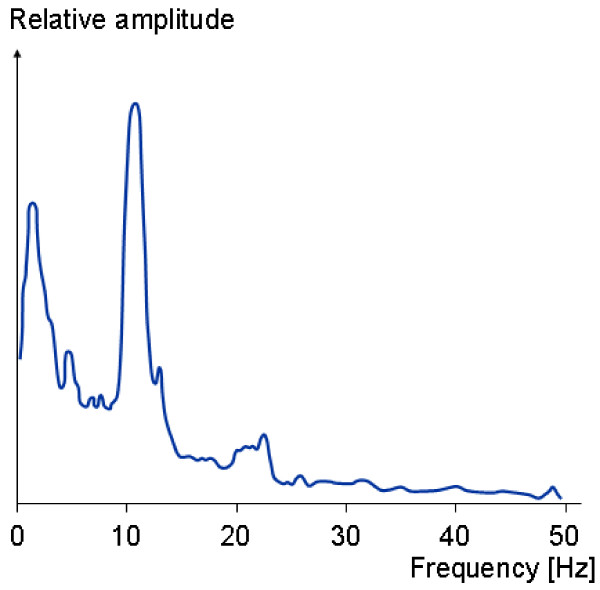
The magnitude spectrum of a normal EEG signal.

The EEG signal corresponds to the electrical potential due to brain (neuron) activity, and can be acquired on the scalp (signal amplitude usually under 100 *μV*) or directly on the cortex (called Electrocorticography – ECoG), the surface of the brain (signal having about 1 *- *2 *mV *of amplitude). The frequency band of normal EEG signals is usually from a little bit above DC up to 50 *Hz *(see Figure 2).

Although EEG signals were initially used just in Neurology and Psychiatry, mainly to diagnose brain diseases as epilepsy, sleep disorders and some types of cerebral tumors, many research groups are now using them as a communication channel between a person's brain and electronic machines, in order to develop systems to improve his life condition. The main point of this idea is the Human-Machine Interface (HMI), also called a Brain-Computer Interface (BCI), a system capable to acquire the EEG signal, to extract features there embedded, to "understand" the intention manifested by the user and to control electronic devices such as a PC, a robot or a wheelchair.

In addition, if the objective is to develop a portable and embedded BCI, low cost, small size, small weight and portability are very important advantages of systems based on the EEG signal when compared to other ways to register brain activity [4]. Other advantages of using EEG signals are: they have good temporal resolution and allows extracting features enough to control electronic devices (since appropriate signal processing methods are used).

**Figure 3:**
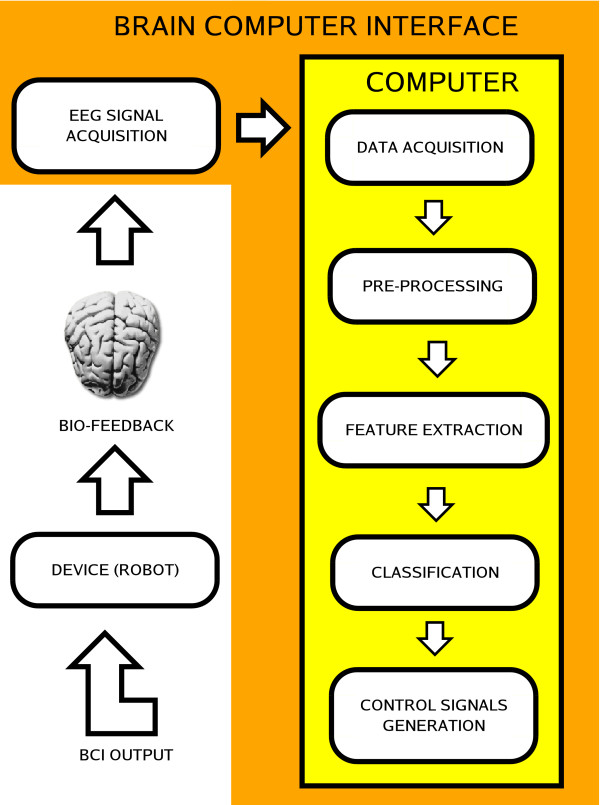
Basic structure of a BCI.

A BCI, as a HMI, follows the basic structure presented in Figure 3, which is composed of two main parts. The first one is responsible for acquiring the signal and for conditioning it (by filtering and amplifying it). Following, the analog signal just acquired is converted to a digital one (A/D converter), which is delivered to a PC. The second part of the BCI starts with a pre-processing algorithm, necessary to remove undesirable signals, called artifacts, usually corresponding to signal levels much higher than the studied ones. After such feature extraction, the system has information enough to make decisions (classify) and generate the necessary control actions to be delivered to the electronic device to be controlled. The user, in such a diagram, closes the bio-feedback link.

**Figure 4:**
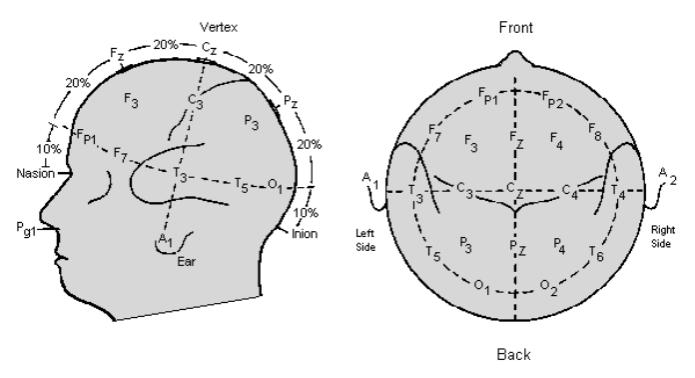
The 10–20 International System for placing electrodes.

The information extracted from EEG signals, in this work, is related to ERD (Event Related De-synchronization) and ERS (Event Related Synchronization), which appear in the alpha band (8 to 13 *Hz*) of the EEG spectrum. They are event-related phenomena corresponding to a decrease (ERD) or an increase (ERS) in the signal power (in the alpha band of the EEG spectrum). The ERD and ERS patterns are usually associated to a decrease or to an increase, respectively, in the level of synchrony of the underlying neuronal populations [5]. The EEG signal, in this case, is captured through electrodes placed in the positions *O*_1 _and *O*_2_, in the occipital region of the human head, over the visual cortex, like depicted ahead, in Figure 4.

This work presents a sequence of development for an HMI that takes into account all the previous considerations, and in which the degree of difficulty in both signal acquisition and processing is gradually increased. In such a sequence, in the first stage of the implementation the HMI developed is based on the signal caused by eye blinks (an EMG signal). Such a system was used to control a mobile robot, which was able to navigate in a semi-structured environment. Next, a module capable to acquire and process EEG signals was also implemented, which currently explores the ERS/ERD complex of the EEG signal acquired by two electrodes placed in the occipital region of the head of an individual with motor dysfunction (such a signal is related to visual activity). Such modules have been used to control a mobile robot and a robotic manipulator, respectively. Experimental results using such modules are presented in the paper, as well as some discussion about the future of the research our group is developing is presented.

### Brief review on commanding a mobile robot using EMG signals

EMG (ElectroMyoGram) signals are generated by the contraction of the human-body muscles. They are currently being used to command robotic devices like manipulators (robotic arms and hands) and mobile robots (robotic wheelchairs). The goal is to develop systems capable to help people with different motor disabilities.

The systems shown in [6] and [7] allow controlling robot manipulators through some muscular signals. In [6], specifically, the left and right Flexor Carpi Radialis muscle (a muscle near elbow) are used, with the third sensor placed on the Brachioradialis muscle (a muscle on the forehead), to generate a series of activations to open/close a gripper, and to move it to pre-defined positions, thus allowing people with severe motor disability to execute activities of daily life.

In [8], EMG signals are acquired from biceps brachii, the muscle that is the main responsible for the flexion of the elbow of an individual, to teleoperate a robot arm. Although the dynamic model of the robot arm is taken into account, the experimental results there presented have just shown the robustness of the system when regarding smooth elbow movements. A similar work is presented in [9]. However, in this last one, the experiments conducted show the system accuracy and robustness for both slow and fast catching motions. In addition, experiments with targets being placed in different directions and distances are also conducted. An EMG-based command is also used in a dexterous robot hand in [7]. The system reproduces the finger motions when the user moves his/her fingers, and can be teleoperated as in [8]. The experimental success rate for six different types of finger motions reached more than 77%.

Some systems use EMG-based signals for commanding robotic wheelchairs. A robot wheelchair is useful for people with motor disabilities in both lower and upper extremities, due to paralysis or amputation. In [10] three solutions are presented to set the wheelchair in motion: an HMI based on EMG signals, face directional gesture, and voice. The EMG signals are acquired from the elevator scapulae muscle, and can be generated by voluntary elevation movements of both left and right shoulder. The experimental results shown in [10] allowed concluding that the system can be used by people with motor disabilities, although just indoor experiments have been performed. Another conclusion presented in [10] is that it is necessary to build an environment map to perform long-time outdoor navigation.

In [11] and [12] systems very similar to those proposed in [10] are presented, also using commands based only on EMG signals. The great advantages of the system proposed in [11], however, are its low cost and its small size, which are due to the use of a non-commercial EMG amplifier. In addition, in [12] it is used a combination of the movements of the muscles of the shoulder and the neck to command the wheelchair. In the several works which address the command of robots through systems based on EMG signals, many types of muscles are used as command signal generators. In general, the upper extremity muscles, e. g., the muscles for wrist and elbow flexion, are the most commonly used. When the individual does not have such muscles, however, it is common to use the shoulders and/or neck motion muscles. Sometimes, when the individual can not move any part of his/her body, but he/she can blink his/her eyes, the EMG signals can still be useful for commanding devices. In such cases, as addressed here, the EMG signal is generated by blinking the eyes.

### Brief review on commanding a robot using EEG signals

The electrical potential caused by the neuronal activity, recorded from the scalp (a non-invasive way) or directly from the brain cortex (ECoG), can be used to control robots and other electronic devices. In the sequence, some meaningful works dealing with such subject are commented, in order to provide a brief overview about brain-actuated devices.

Example of ECoG recording can be found in [13]. The electrical activity acquired on the brain cortex surface is not attenuated as the signal captured on the scalp (after crossing the cranium), thus presenting a better quality. The objective is to map the data corresponding to the multi-channel neural spikes of a monkey to the 3D positions of its arm positions. The predicted position of the hand of the monkey is used to control a robot arm.

A brain-actuated control of a mobile robot is reported in [2]. Two individuals were able to control a small Khepera mobile robot navigating through a house-like environment after a few days of training. EEG potentials were recorded through eight electrodes placed on standard fronto-centro-pariental positions, in a non-invasive way. Spatial filtering, Welch periodogram algorithm and a statistical classifier were used to recognize mental tasks, such as "relax", imagination of "left" and "right" hand (or arm) movements, "cube rotation", "subtraction", and "word association", which were used by a finite state automata for controlling the robot. An asynchronous BCI was adopted, which avoids the waiting for external cues, unlike a synchronous one. A meaningful rate of correct recognition (above 60%), associated to an error rate below 5%, was obtained with such a BCI, which resulted in a brain-actuated control of the robot demanding no more than 35% of the time spent for manually controlling the robot, for both individuals. A similar work is reported in [14], in which a virtual keyboard and a mobile robot are controlled by using an asynchronous BCI, which was tested by 15 individuals.

Most recent studies have shown that dissatisfaction of individuals can be used to correct machine errors. When an individual sends a command to a device and gets a non-expected response, the awareness of erroneous responses, even when the error is not made by the individual himself, can be recognized in the brain signal captured. This is done through error-related potentials (ErrP) and is used to improve the performance of the BCI [15].

Several works reporting the use of the signal caused by brain activity to command devices have been published. However, the Human-Machine Interfaces or Brain-Computer Interfaces used are still too much expensive. In some cases, they are even more expensive than the robot, the wheelchair or other device being commanded. Regarding this topic, the HMIs proposed in this work are attempts to get a good compromise between effectiveness for the application and cost.

## Methods

Experiments based on muscular and cerebral activities are here accomplished in order to verify that a human operator is capable to command robots through Human-Machine Interfaces. Two HMIs, based on different electro-biological signals were developed, namely an EMG-based HMI and an EEG-based HMI. The first one allows a person to command devices through the signal generated by blinking his own eyes [16]. The other one allows decoding brain commands as well as controlling devices like robots [17]. In this section a brief introduction to such systems is presented.

### An EMG-based human-machine interface

**Figure 5:**
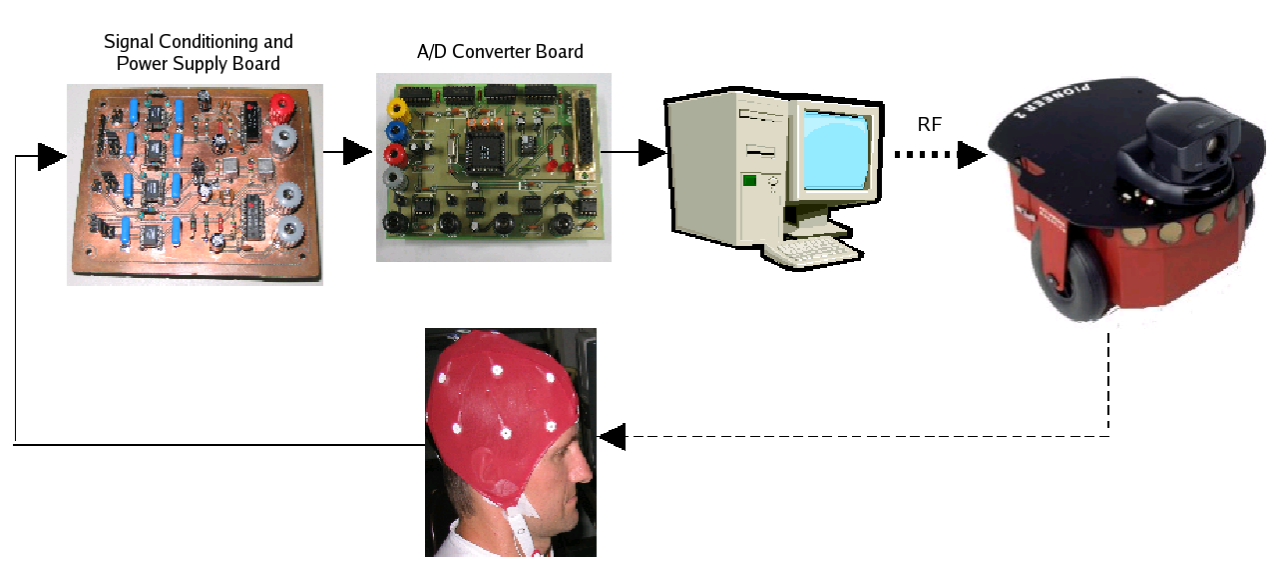
The structure of the proposed HMI.

Figure 5 shows the structure of the EMG-based HMI developed to allow controlling a mobile robot. It is composed of a signal acquisition and a signal processing subsystems. No complex preparation is required when an individual is asked to use such HMI to control a device. He is supposed to use a commercial cap (just for convenience) with the electrodes correctly placed, according to the 10–20 International System (see Figure 4). The head positions to be used should be clean, not being necessary to shave the hair. On the other hand, it is necessary to apply a gel between the electrodes and the scalp, in order to match the contact impedances. A reference electrode should be connected to the left or the right ear.

After being correctly dressed, the cap should be connected to the signal filtering and amplification subsystem. The amplification board embeds a power source that is designed to reduce any spurious interference at the same frequency of the electric appliances or interference coming from other external electronic equipments, such as switching mode power supplies, on the acquisition system. Then, the signal filtering and amplification subsystem is connected to the A/D conversion subsystem. Four analog channels are available in such A/D conversion subsystem, which allow expanding the signal acquisition capacity through cascade connections, thus increasing the number of channels being processed. After establishing such connections, the digital data delivered by the A/D converter is sent to a desktop computer, through a DB9 serial cable.

Then, the system is now operating: the user's electro-biological signal is acquired by electrodes that send it to the signal filtering and amplification subsystem. Afterwards, this signal is sent to another board to be converted to digital data. Finally, such signal is transmitted to a desktop computer, where it is processed to generate (or not) a specific command for controlling a mobile robot. The user of the HMI closes the control loop, providing the necessary biological feedback.

The interface for the user-machine communication is programmed in the desktop computer, as well as the signal processing software that sends the control commands to the mobile robot. These commands are transmitted to the robot through an Ethernet Radio.

The experiments here reported were carried out using a Pioneer 2-DX nonholonomic wheeled mobile robot. This robot has a microcontroller for low level instructions, and an embedded PC (Intel Pentium MMX 266 MHz, 128 MB RAM) for high level tasks like sensing and/or navigation.

**Figure 6:**
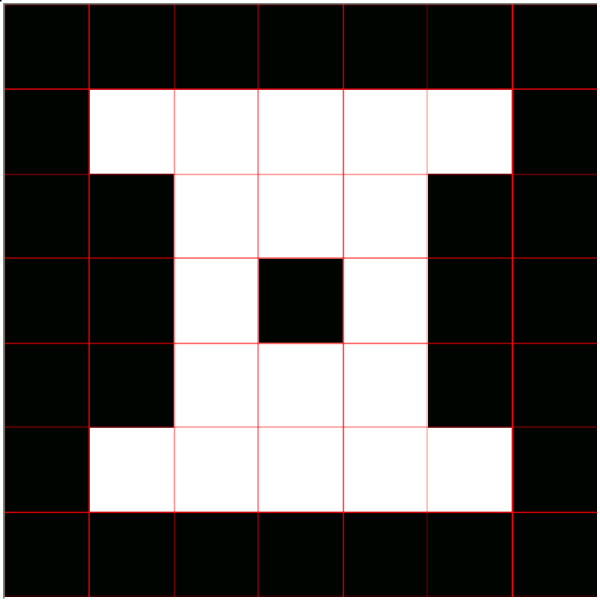
The electronic board used to represent the robot working space.

For generating a command, the user should be able to blink his/her eyes. From the eye-blinks a command is decoded and transmitted to the mobile robot, which is commanded to go from a site to another site in its working environment. To help the user in the task of guiding the robot through its working environment, an electronic board with automatic scanning was implemented (in the desktop microcomputer). Such a board represents the area of the robot working environment, divided in cells, like it is depicted in Figure 6. This way, when the cell the user wishes to command the robot to go to is swept, he blinks a determined eye and the corresponding EMG signal is captured and processed by the signal acquisition and processing subsystems.

**Figure 7:**
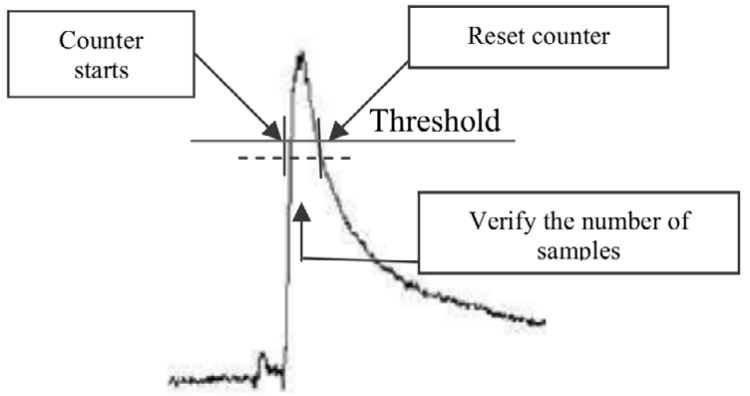
The eye-blink detection scheme.

Since the EMG signals due to eye blinks have a well-defined standard behavior, like it is presented in Figure 7, the necessary processing system is relatively simple. It works as follows: firstly, a threshold is experimentally established for each user, based on the changes observed in a signal interval that contains a set of eye blinks (training stage). During the system run, whenever the signal generated by an eye blink goes above such a threshold, a counter starts counting the number of samples received ever since. When the signal falls below the threshold, the number of samples counted is compared with a predefined one: if it is greater than the pre-defined number, the HMI detects an eye blink. Otherwise, the HMI detects that there was not an eye blink. This means that only eye-blinks whose time-duration is greater than a certain number of sampling intervals is considered as effective eye-blinks. After that, the counter is reset, and a new cycle starts.

### EEG-based human-machine interface

**Figure 8:**
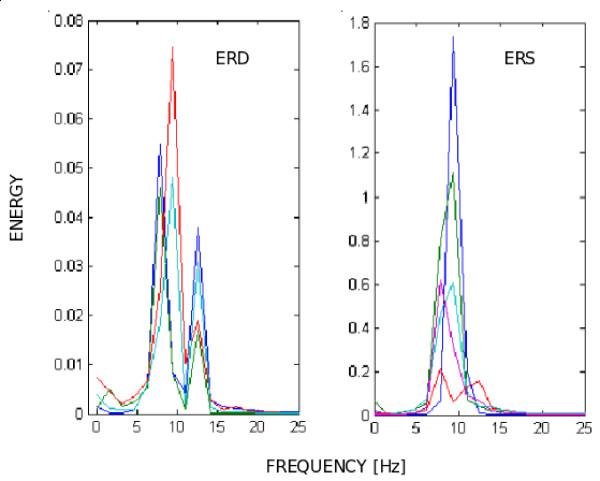
ERD and ERS observed in alpha band.

**Figure 9:**
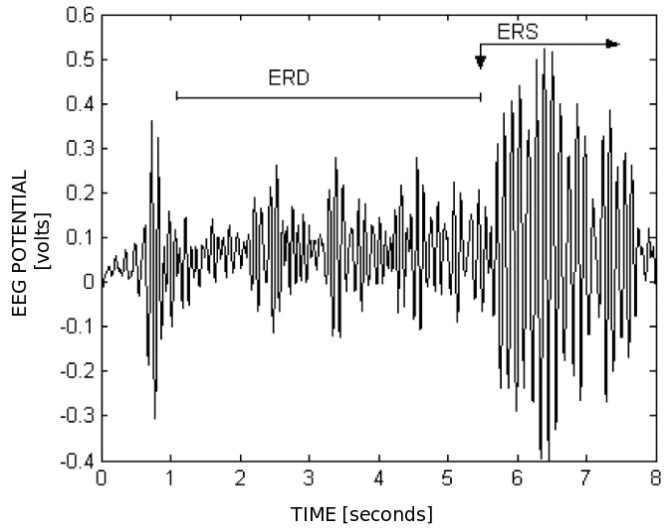
Energy increase during an ERS.

Looking into the alpha frequency-band, for an EEG signal captured over the occipital region of the user's scalp, any increase and decrease of signal power can be detected. The occipital region is responsible for processing visual information, in such a way that in the presence of a visual stimulus (eyes opened) the signal power in the alpha band decreases, characterizing an ERD. On the other hand, if the eyes are closed, the human operator has his/her visual area relaxed, with a few or even none visual stimulus, characterizing a high signal power, which corresponds to an ERS. As presented in Figure 8, the power of an ERS can be many times the power of an ERD. A threshold (5 to 10 times the value of an ERD) can be established to detect an ERS. Figure 9 shows the energy increase associated to an ERS. It is important to mention that EEG levels change constantly, thus requiring a calibration step to detect the basic ERD level before starting the analysis. These two states (power increase and power decrease) can be associated to actions such as "select the current symbol of the table". In order to validate this idea, experiments with robots were accomplished, and the results are reported here.

Additional attention should be given to artifacts. Eye blink, cardiac rhythms, noise coming from the 50–60 Hz power line and body movement are examples of artifacts. They can mask the studied signal and should be avoided and removed. The frequency band explored here is from 8 to 13 Hz, and with a bandpass filter it is possible to remove artifacts due to eye blinks, which usually occurs between 0.1 and 5 Hz, as well as the noise of 50–60 Hz coming from the power line [18,19].

The BCI adopted here to extract information on the occurrence of the ERS/ERD events is relatively easy to use. As in the EMG-based case, shaving the operator's head or other special preparation is not necessary. However, a gel is used to improve the contact between the electrodes and the skin. The electrodes are placed in the positions *O*_1 _and *O*_2_, like illustrated in Figure 4, with the reference connected to an ear lobe (according to the 10–20 international system of electrodes positioning).

Such a BCI was tested by a group of 25 individuals (from 20 to 50 years old), some of which had suffered cases of meningitis or epilepsy. Three stages of experimentation were accomplished: in the first one, the operator uses an event detector that recognizes the states of high and low energy of the acquired signal; in the second one, the operator is invited to command the robot in a simulation environment, and, in the last one the operator applies what he learnt in the two previous stages to command a real robot [1].

An operator is considered capable of having full control of the BCI if he succeeds in the first and second stages, what means if he showed to be able to command the robot in a simulation environment using the BCI.

Two experiments were carried out to validate the BCI and the control scheme as a whole. In the first one, the operator used the BCI to guide a mobile robot in an indoor structured environment, thus emulating a wheelchair taking the operator to the rooms of a house or office, for example. In the second one, the operator uses the BCI to command a manipulator, emulating a prothesis or an orthosis, including the teleoperation via a TCP/IP channel.

#### First experiment: commanding the mobile robot

**Figure 10:**
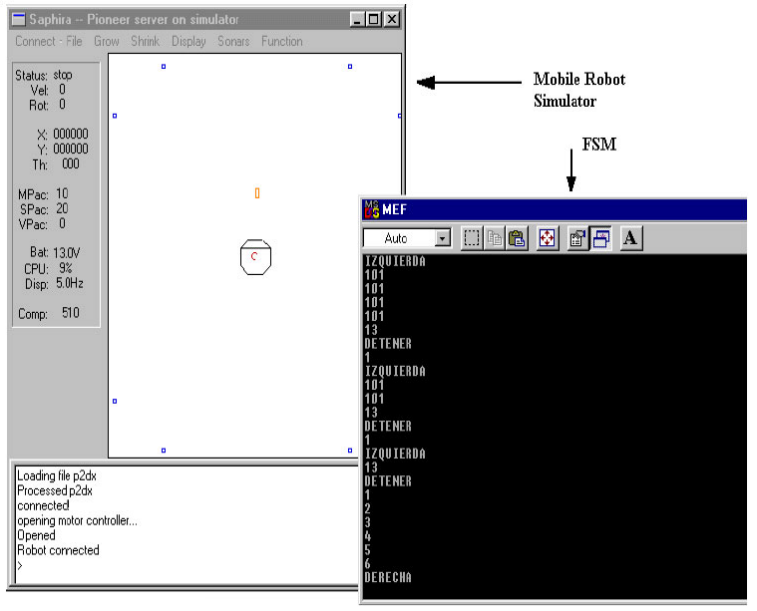
Simulated environment in which the mobile robot navigates.

**Figure 11:**
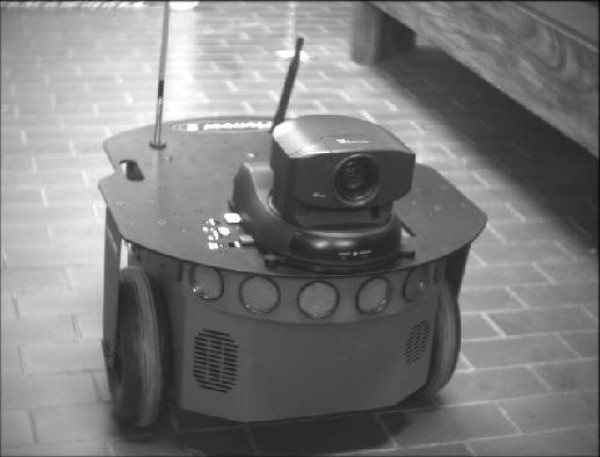
Pioneer 2-DX robot operated through EEG signals.

The BCI so far discussed was used to operate a mobile robot Pioneer 2DX in a simulated environment (Figure 10) and in a real one (Figure 11). The analysis of the signal power in the alpha frequency band was used to change the states of a Finite State Machine, generating commands such as go ahead, turn right, turn left and go back to the mobile robot.

#### Second experiment: commanding the manipulator

**Figure 12:**
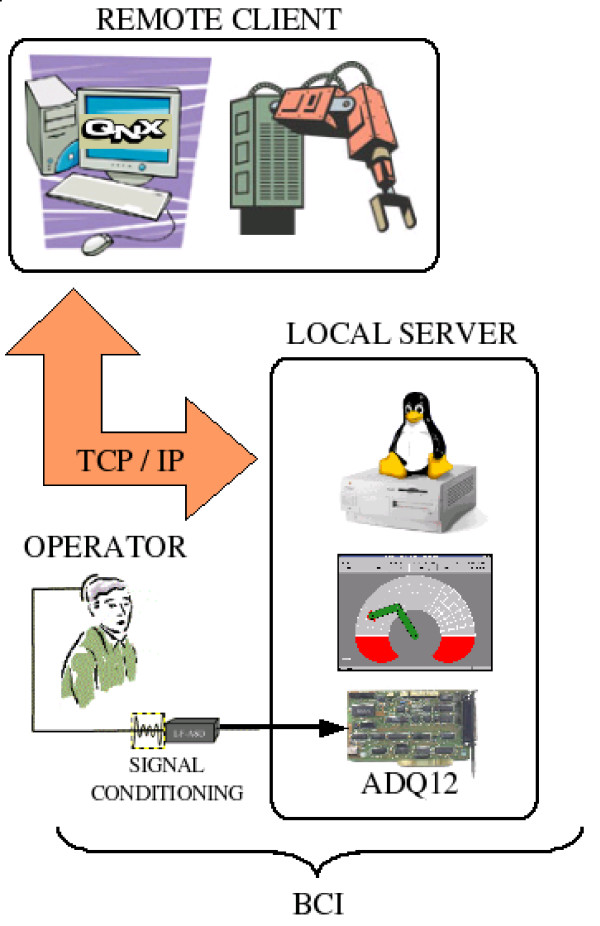
Illustrating the whole system.

**Figure 13:**
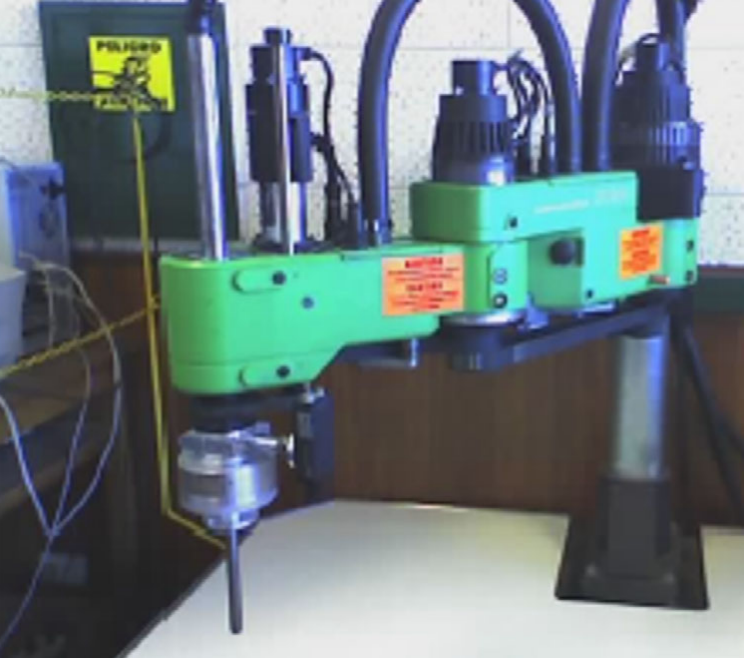
The manipulator Bosch SR-800.

**Figure 14:**
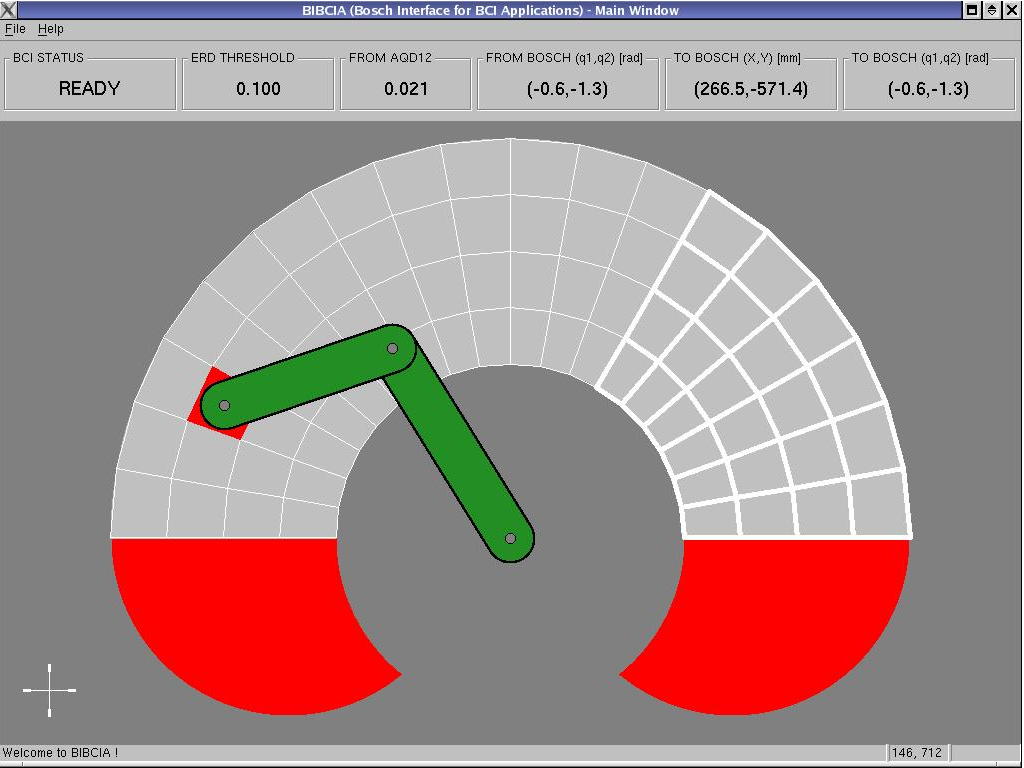
The local Graphical Interface.

Figure 12 illustrates the experiment accomplished. In the case of operating a manipulator (BOSCH SR800 – Figure 13) via TCP/IP, it is presented to the operator the manipulator's workspace divided in cells. The application scans all cells and the analysis of the signal power of the user's EEG signal in the alpha frequency band is used to select one of them. The selection is done when an ERS pattern is recognized. When it is done, the coordinates of this cell are sent, through a TCP/IP channel, to a remote computer in charge of controlling the manipulator, moving its end effector towards the desired position. At the same time, the data incoming from encoders are sent back to the user's PC (the local computer) in order to update the screen with the current positions of the manipulator. Figure 14 presents the graphical interface used by the operator to select the desired position.

It is important to remember that in both cases a calibration process is necessary before starting the experiments. This procedure consists of acquiring about 10 seconds of EEG data to analyze the ERD level. Based on this information, the threshold used to detect an ERS is set to 5 up to 10 times the level corresponding to an ERD. This is very important because these levels change constantly in time and from an individual to another.

## Results and discussion

Both HMIs have been used to command robotic devices by individuals previously trained to operate them. The EMG-based HMI was used to command a mobile robot, while the EEG-based HMI was used to command a mobile robot and a robotic manipulator as well. In this section, the results of each test accomplished are reported and discussed.

## EMG

Firstly, eight volunteers were asked to accomplish ten eye blinks with each eye, in order to test the eye blink identification algorithm. The results of these experiments are shown in Table 1, just for the volunteers who were able to blink both eyes.

**Table 1:** Success rates blinking the right and left eyes

Volunteer	Right Eye	Left Eye
1	8	9
2	10	10
3	10	8
4	10	10
5	10	9
6	10	10
7	10	10

The main result obtained is a rate of positive identification of the eye blinks about 95.71% of the cases of volunteers with the ability to blink both eyes, which allowed concluding the viability of using the system to command devices.

One out of the eight volunteers that presented a good performance in the experiment with the eye blinks-based system was asked to determine a destination point on the electronic board. After the volunteer selected a destination point through eye blinks, the control software started to guide the robot to such point, following the path determined by a path planning algorithm [16], which is based on the Dijkstra's Algorithm, that determines a secure path (more distant of walls and obstacles).

**Figure 15:**
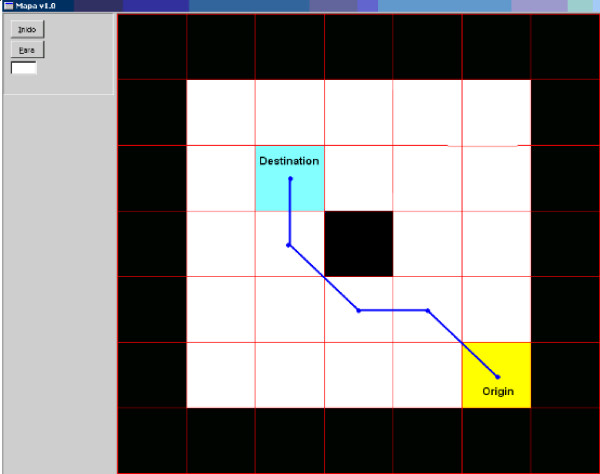
The path generated by the system.

**Figure 16:**
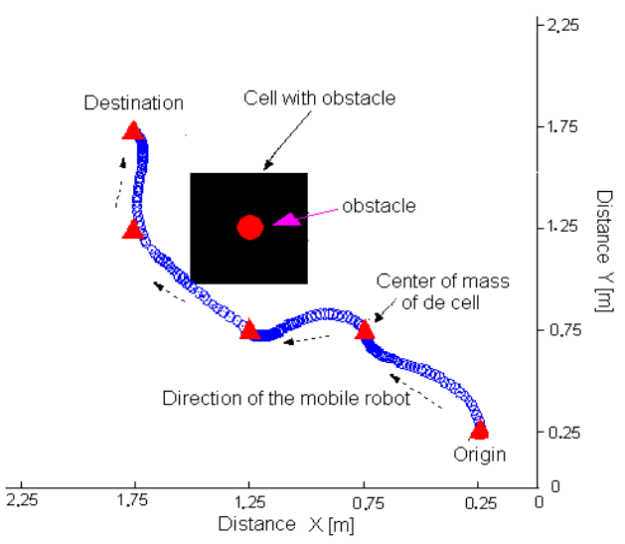
Result of the experiment with unpredicted obstacle distant of the robot's navigation path.

Figure 15 shows the map of a navigating environment and the path generated by the system to go from an initial position to a destination position selected by the user. That path is transmitted to the mobile robot, which knows its navigating environment. Figure 16 shows the result of the navigation executed by the robot during such an experiment.

### EEG

**Figure 17:**
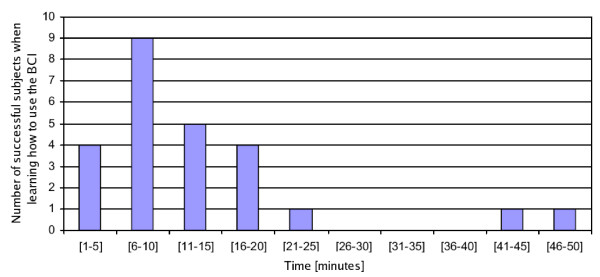
Number of individuals that managed to learn how to use the BCI versus the training time required (in minutes).

All the 25 individuals invited to test the system learnt how to use the BCI in just one training session. The statistical results presented in Figure 17 show the average training time necessary to use the BCI and to learn how to manage the mental states associated to concentration and relaxation of the visual area of the brain. As it can be seen, most of the individuals learnt how to use the BCI in less than 15 minutes with just one experiment. The minimum and maximum training times observed were 3 and 50 minutes, respectively. Even though some of the individuals that carried out the experiments had suffered cases of meningitis and epilepsy, the BCI has not been tested by people with severe neuromotor problems so far. These tests are very important, taking into account that these are the individuals supposed to use this technology. The results presented here show the versatility of the BCI when used to control robotic systems. The short training time required to operate it and its low cost are other meaningful features.

Although for the applications here addressed the EEG-based BCI so far discussed have run and performed very well, more natural mental states, such as thinking about moving a right hand in order to move the robot to right, for example, should be more interesting. More mental states provides more flexibility when connecting them, or a combination of them, to actions to be performed by mechanical devices. These topics are currently being addressed by our research group.

Nevertheless, an analysis of the experiments so far accomplished, namely guiding a mobile robot and controlling the positioning of the end effector of a manipulator, shows that the BCI so far adopted has proven to be effective to command robotic systems, including remotely.

## Conclusion

Two different HMIs were here developed to allow an operator to control a robot without using his hands. He used only the EMG signal generated by blinking his eyes, or the EEG signal generated by intercalating states of concentration and relaxation of the visual cortex of his brain (visual stimuli). In both cases, the HMIs have proven to be of simple implementation and of low cost, besides exhibiting good performance. The EMG signal was chosen as electro-biological signal due to the fact that it is a well behaved signal easily acquired and processed, in comparison to other electro-biological signals, as the EEG signal, for instance. The results demonstrate that such a HMI is easy to handle by users who can blink their eyes according their wishes. This HMI was tested in controlling a mobile robot: an experiment in which a user should select a final destination to which the robot is supposed to go to, through a suitable sequence of eye blinks, which should be reached by the robot. In all tests, the mobile robot effectively reached the destination selected by the user.

The EEG-based HMI can be seen as an evolution of the EMG-based HMI due to the increase in the degree of difficulty of both the acquisition and processing subsystems. It has been used the so-called ERS/ERD complex, which can be identified in a relatively easy way, which provided, in two cases presented (a mobile robot guidance example and a robotic manipulator control example), a simple and low cost solution. In both cases presented, the desired commands were effectively executed by the robotic devices.

The work so far reported are the beginning of the development of a system intended to assist people suffering of neuromotor diseases, including people with severe dysfunctions. The next steps are to convert a commercial wheelchair in an autonomous mobile vehicle; to implement the HMI on board such autonomous wheelchair to assist people with motor diseases; to explore more characteristics of the EEG signal, in order to make the Brain-Computer Interface (BCI) more robust and faster, thus allowing its secure use by people with severe motor dysfunctions.

## Competing interests

The author(s) declare that they have no competing interests.
